# The Brief Prakriti Inventory: Latent structure, reliability, and validity

**DOI:** 10.12688/wellcomeopenres.25166.1

**Published:** 2025-12-10

**Authors:** Hemant Bhargav, Umesh Chikkanna, Bharath Holla, Rama Arya, Rushali Daga, Nishitha Jasti, Sadavrat Amlani, Chandrasenan Santhosh, Vidhya Sanker, Akhila Soman, Krishnaja Unnikrishnan, Venkataram Shivakumar, Shivarama Varambally, Kishore Kumar Ramakrishna

**Affiliations:** 1 Integrative Medicine, National Institute of Mental Health and Neurosciences, BENGALURU, Karnataka, 560029, India; 2 Sports Science and Yoga, Ramakrishna Mission Vivekananda Educational and Research Institute, Belur Math, West Bengal, 711202, India

**Keywords:** Prakriti, Ayurveda, Personality Assessment, Traditional Medicine, Psychometrics, Individual Differences

## Abstract

**Background:**

Ayurveda, the traditional Indian medicine system, conceptualizes individual personality (Prakriti) through three dimensions, Vata, Pitta, and Kapha, based on physical, physiological, and psychological traits. Existing tools for Prakriti assessment often lack robust psychometric validation and accessibility. We developed and validated the Brief-Prakriti Inventory (BPI), a 21-item self-report instrument for assessing traditional Indian personality concepts.

**Methods:**

An initial 30-item pool was derived from classical Ayurvedic texts and contemporary literature, covering three domains. Following pilot testing and psychometric screening, 21 items were retained. Items used nominal response formats, each mapped to a dosha, with randomized option order via REDCap. Psychometric evaluation employed Multiple Correspondence Analysis (MCA), Latent Class Analysis (LCA), and Item Response Theory (IRT) in a community sample (N = 1857). Validity was assessed via test–retest reliability, convergent validity with traditional AYUsoft assessments, and divergent validity using Western personality traits (Mini-IPIP).

**Results:**

MCA revealed distinct dosha-aligned item clustering, particularly among participants with dominant dosha profiles ( Figure 1). LCA supported a three-class model (dominant-only: entropy R2 = 0.96) ( Figure 2, Supplementary Figure 1). IRT analyses showed strong fit (CFI = 0.967, RMSEA = 0.023) and good reliability (Vata = 0.87, Pitta = 0.75, Kapha = 0.87) ( Figure 3). Psychological items showed highest discrimination; physiological items displayed higher difficulty thresholds. Test–retest reliability was high (ICCs 0.83–0.90). BPI subscales correlated strongly with traditional assessments (r = 0.78–0.84) (Supplementary Figure 2) but minimally with Western personality traits ( Figure 4), supporting construct distinctiveness.

**Conclusions:**

The BPI is a brief, reliable, psychometrically validated self-report tool that captures latent dosha typology consistent with Ayurvedic theory. By grouping individuals into Prakriti-based clusters, the BPI will enable biological phenotyping of dosha-linked variability and support personalized, culturally contextualized interventions in integrative and mental health care.

## Introduction

Prakriti is an Ayurvedic constitution-based framework describing stable individual differences that integrate psychological tendencies, bodily form, and physiological regulation. Individuals are typically classified into three core profiles (Vata, Pitta, Kapha), each reflecting coherent patterns in activity and reactivity, appetite and digestion, sleep–wake rhythm, energy levels, and affective–behavioral styles. These profiles function as concise multidomain phenotypes rather than isolated traits, positioning Prakriti as complementary to mainstream personality models ( Prasher *et al.*, 2008; Sethi *et al.*, 2011).

Most contemporary personality models, such as the Five-Factor Model, focus on psychological dispositions and their behavioral manifestations. They do not directly capture enduring somatic characteristics such as body build, homeostatic tendencies such as thermal sensitivity, or routine physiological patterns such as appetite and sleep architecture, all of which are integral to Prakriti descriptions ( Delle Fave *et al.*, 2015). Prakriti can therefore be conceptualized as a cross-domain phenotype that integrates temperament-like tendencies with stable physical and physiological attributes. This distinction has empirical significance. Although Big Five traits are strong predictors of many psychosocial and health-related outcomes, they account for relatively little variance in individual differences in bodily regulation. Multidomain constructs such as Prakriti may therefore provide additional explanatory value by capturing variance not represented in purely psychological measures ( Rizzo-Sierra, 2011).

Other cultural systems have also proposed constitutional typologies, such as Sheldon's somatotypes (ectomorph, mesomorph, endomorph), Kretschmer's pyknic and leptosomic builds, and Korean Sasang medicine’s Tae-Yang, So-Yang, Tae-Eum, and So-Eum types. Like Prakriti, these frameworks aim to link stable constitutional profiles with psychological tendencies and health risks, though they vary in operationalization and empirical support ( Rizzo-Sierra, 2011).

A validated, multidomain Prakriti measure has both theoretical and applied value ( Bhargav *et al.*, 2021; Mukerji, 2023). Theoretically, it would enable examination of how constitutional dispositions in bodily regulation relate to temperament and broad personality spectra, clarifying areas of convergence and divergence. Applied, such a measure could be used to test whether constitutional profiles account for variance in stress responsivity, symptom expression, or treatment adherence beyond standard personality traits, thereby informing integrative models of mental and physical health. Early studies have demonstrated molecular and physiological differences among individuals with extreme (single-dominant) Prakriti profiles at the levels of gene expression, genetic variation, epigenetic signatures, and autonomic responses ( Aggarwal *et al.*, 2010; Govindaraj *et al.*, 2015; Prasher *et al.*, 2008; Rani *et al.*, 2022; Rotti *et al.*, 2014; Sethi *et al.*, 2011; Tiwari *et al.*, 2017). These findings motivate the development of transparent, psychometrically defensible measures that can support cumulative tests of external relations in health and behavior.

Despite growing interest, measurement approaches remain fragmented. Over the past decade, operationalization attempts have included clinician ratings, proprietary software (e.g., AyuSoft), and machine learning models on lengthy self-report questionnaires ( Gupta *et al.*, 2025). These tools vary in sampling, operational definitions, and reporting standards, and few apply latent-variable models suited to categorical data. No brief, openly described, psychometrically validated instrument exists for mainstream psychological research.

To address this gap, we developed and validated the Brief Prakriti Inventory (BPI), a 21-item self-report instrument refined from a larger pool grounded in classical descriptions and prior tools ( Table 1, Supplementary Tables 1–3). The validation strategy followed current best practices for categorical data: (i) Multiple Correspondence Analysis (MCA) to examine low-dimensional structure; (ii) Latent Class Analysis (LCA) to identify potential typological groupings; and (iii) nominal-response Item Response Theory (IRT) to estimate item discrimination, information, and score precision, guiding refinement. Temporal stability and external validity were assessed through test–retest reliability, convergent validity with a clinician-guided assessment (AyuSoft), and divergent validity with the Mini-IPIP to confirm distinctiveness from Big Five traits. This design enabled us to determine whether a brief, self-administered instrument could reliably recover latent regularities corresponding to Vata, Pitta, and Kapha, and relate them to established constructs in expected but nonredundant ways. By combining latent-structure analyses with item-level modeling and comprehensive reliability and validity testing, the BPI provides a concise, empirically grounded measure suitable for replication, cross-cultural personality research, and studies of clinical or biological correlates under standardized conditions.

**Table 1.** Brief Prakriti Inventory Item Pool: Initial 30-item pool after expert consensus and literature review. The final 21-items retained after psychometric evaluation are shown in bold font. Initial 30-item pool after expert consensus and literature review. The final 21-items retained after psychometric evaluation are shown in bold font.

Items	Physical	Physiological	Psychological
**1**	Body frame	**Digestion**	**Emotional temperament**
**2**	**Skin texture**	**Appetite**	**Emotional arousal**
**3**	**Hair texture**	**Thirst**	**New learning and memory**
**4**	Eye characteristics	**Sleep patterns**	Communication style
**5**	**Perspiration**	**Body weight changes**	Decision-making
**6**	**Body Movements**	**Bowel movements**	Mood rhythms
**7**	Facial features	**Temperature sensitivity**	**Competitiveness**
**8**	Teeth	**Taste preference**	**Attention and concentration**
**9**	Tendons and Veins	**Physical endurance**	**Work initiative**
**10**	Walking	**Infection resistance**	**Speech Patterns**

## Methodology

### Study design

This cross-sectional, online survey aimed to develop and validate the BPI, a self-report instrument assessing multidomain Prakriti profiles.

### Sample size estimation

Sample size requirements were determined from psychometric analysis guidelines. A minimum of 10–20 respondents per response option per item was targeted, corresponding to ~1000–1500 participants for reliable IRT analyses. The achieved sample size (N = 1857) exceeded this threshold, providing sufficient power for IRT and LCA. Subsamples for test–retest reliability (n = 116), convergent validity (n = 104), and divergent validity (n = 541) met or exceeded the required size to detect medium correlations (r = 0.3) with 80% power at α = 0.05.

### Participants and recruitment

Participants were recruited between June 2023 and June 2025 through online advertisements, university postings, and snowball sampling. Inclusion criteria were: (1) age 18–65 years, (2) ability to read and understand English, and (3) no diagnosis of severe mental illness or cognitive impairment that could interfere with questionnaire completion. Exclusion criteria included >10% missing responses or duplicate entries identified via IP tracking.

The final sample had a mean age of 30.8 years (SD = 11.4), 66.9% female, and educational backgrounds ranging from primary or less (0.8%), high school (3.3%), bachelor's degree (44.8%), to postgraduate degree or more (51.1%).

### Instrument development

An initial pool of 30 items was generated from classical Ayurvedic texts and modern literature, covering physical, physiological, and psychological domains ( Table 1, Supplementary Table 1). Each item offered three nominal options corresponding to Vata, Pitta, or Kapha, based on characteristic properties (Gunas) (Supplementary Table 2). A pilot study with 150 participants informed item reduction using Multiple Correspondence Analysis (MCA) and item performance metrics, yielding a 21-item inventory ( Table 2; Supplementary Table 3).

**Table 2.** Validated Final Version of Brief-Prakriti Inventory (BPI-21): Item-wise Response Options. In this 21 item questionnaire. Each item contains three options corresponding to Vata, Pitta, and Kapha, of which participants select one. Responses are coded such that the chosen dosha for each item receives a score of 1, while the unselected doshas receive 0. Raw scores are obtained by summing responses across all 21 items, yielding three totals (Vata, Pitta, and Kapha), each ranging from 0 to 21. To facilitate interpretation, raw totals are converted to percentages by dividing the score for each dosha by the maximum possible score (21) and multiplying by 100. General Instructions 1.   This questionnaire helps you understand your unique constitution (Prakriti) according to Ayurveda. It explores your physical, physiological and psychological characteristics to determine your Prakriti. 2.   Read each question carefully. There are no right or wrong answers, as everyone is unique. 3.   The options are presented in no specific order (When presented with REDCap) 4.   Choose the option that best reflects your natural tendencies and usual patterns. 5.   Answer honestly and intuitively. 6.   If you find yourself relating to multiple options, choose the one that has more characteristics that resonate with you. 7.   Trust your gut feeling when selecting an option.

BPI Item	Option 1: Vata	Option 2: Pitta	Option 3: Kapha
1) Skin texture	My skin is generally dry, or rough, especially in areas like the forearm, elbows, and knuckles. Lotions and oils absorb very quickly and the skin dries up.	My skin is generally soft and supple, warm to the touch. I tend to develop pigmentation, dark spots, pimples, or moles easily.	My skin feels oily and clear. Lotions and oils take a while to absorb.
2) Hair	My hair tends to be dry, brittle, rough, and may have split ends.	I have medium-density hair that's naturally straight to slightly wavy. It can be prone to premature graying and thinning.	My hair is dense and smooth. It may be straight, wavy, or curly. My scalp tends to be oily.
3) Body Movements	I often move around a lot in my seat, play with my hands or feet when seated, change my position frequently, and find it hard to sit still for long periods.	I'm naturally coordinated and agile.	I generally prefer a calm and slow pace in my daily activities.
4) Physical endurance	I tend to have bursts of energy but tire easily. I may find it challenging to sustain physical activity for long periods.	I have a strong drive and enjoy intense physical activity. I tend to be competitive and push myself to my limits.	I have good stamina and can sustain moderate physical activity for extended periods. I enjoy activities like long walks, but prefer a more relaxed and rhythmic pace.
5) Resistance to Infections	When I get sick, my symptoms can fluctuate quickly. I may feel quite unwell at one point, but then start to feel better soon after.	When I get sick, I often have a strong reaction, like a high fever or inflammation. I usually recover quickly once the illness passes.	I generally don't get sick very often. But when I do, it takes me a while to fully recover, and I might have a lot of congestion or mucus.
6) Hunger/appetite	My hunger fluctuates, it varies from meal to meal. I'm sometimes very hungry and sometimes not hungry at all.	I usually have a strong appetite. I get irritable if I have to wait too long to eat.	I have a good appetite and I can easily skip a meal without feeling overly hungry.
7) Amount of food consumed in a meal	I might eat a large amount in one meal and very little in the next. I often prefer smaller portions and feel full quickly.	I have a strong digestive capacity, my food digests fast and I can handle larger quantities of food.	I have a good digestive capacity, my digestive system processes food at a slow and comfortable pace.
8) Thirst	My thirst is a bit unpredictable. I can go for quite a while without feeling thirsty, then suddenly I feel dry and thirsty. I tend to drink in spurts and generally prefer warm drinks.	I often feel thirsty and prefer cool drinks. I can get uncomfortable if I don't drink regularly.	I generally don't feel very thirsty and can go long periods without drinking. I prefer warm drinks.
9) Body Sweat	I sweat less, and even during exercise, I don't sweat much. My sweat is generally odorless.	I sweat during exercise. My sweat sometimes has a more noticeable odor.	I don't sweat much, and when I do, it usually occurs during intense exercise. My sweat is generally odorless.
10) Memory	I grasp new information quickly but tend to forget it easily. My mind jumps between ideas, and I may struggle to recall things from the distant past.	I grasp new information quickly and I have a strong memory for details. However, I might struggle to remember things if I'm feeling emotional. I can be prone to holding onto past hurts.	I learn and retain information slowly but thoroughly. Once I understand something, I tend to remember it for a long time. I tend to hold onto memories, both positive and negative.
11) Sleep	I generally tend to have a light, restless sleep and wake up easily. My mind often races at night, making it hard to quiet down.	I generally fall asleep easily and have sound sleep. But I can also easily forego sleep if I'm engrossed in a project.	I generally tend to sleep deeply and for long periods. It's hard for me to give up sleep unless absolutely necessary. I sometimes wake up with a stuffy nose.
12) Temperature Sensitivity	I prefer warm environments. I feel okay in moderately hot, humid weather but get uncomfortable in cold temperatures and often need extra layers to stay warm in cold climates.	I prefer cooler environments over warmth and dislike excessive heat or humidity.	I prefer warmth slightly more over cooler environments and can tolerate cold fairly well. I dislike excessive heat or humidity.
13) Initiative to start any work	I have bursts of enthusiasm for new projects, but my motivation can fluctuate, and I might lose interest if things get challenging or monotonous. I enjoy starting new things and thrive on variety. However, I can get bored easily and may struggle to complete tasks that require sustained effort.	I am highly driven and goal-oriented. I take initiative readily and enjoy taking charge. I can be competitive and focused on results. I can get frustrated if things don't progress as expected.	I have a slow and steady approach to work. I need time to gather momentum, but once I start, I am persistent and dislike being rushed.
14) How easily do you get emotionally aroused?	My emotions tend to fluctuate, and I get emotionally aroused easily. I am sensitive to the emotions of others and my surroundings.	I have strong and intense emotions. I can be passionate and driven, but also prone to anger or frustration when things don't go my way. I might express my emotions openly and directly.	I tend to have calm and stable emotions. I am generally peaceful, content, and forgiving. I might not express my emotions openly, but I can hold onto them for a long time.
15) Which of the following best describes your typical speech patterns?	I tend to speak quickly and jump between ideas. I can be a lively and engaging speaker, but also get distracted easily. I'm a quick listener, but my mind can wander, and I might miss details.	I speak clearly and directly, with a sharp and focused tone. I can be persuasive and articulate. I'm an attentive listener, but I might get impatient or interrupt when I have something to say.	I tend to speak slowly and calmly, with a soothing tone. I'm a good listener and choose my words carefully.
16) How do you feel about competitive situations?	Dislike and find them stressful	Enjoy competing and perform well under pressure, but find it mentally and physically exhausting	Handle competitive stress with ease and remain calm under pressure.
17) Bowel movements	My bowel movements tend to be irregular. I often experience constipation, gas, or bloating. My stools are often hard, dry, or small.	My bowel movements are usually regular and frequent. I occasionally have loose stools or a tendency towards diarrhea if I eat spicy or oily foods.	My bowel movements are generally slow and easy. I might experience stools that are heavy and sticky. My stools are often well-formed
18) Tendency to Lose Weight	I tend to lose weight easily, even when I'm not trying to. This can happen if I overexert myself, like pushing myself too hard and taking on too many tasks at once.	I find it relatively easy to lose weight when I actively focus on diet and exercise.	I need to put in consistent effort to lose weight. It might take me longer to see results compared to others.
19) How would you describe your mental state?	Restless and easily distracted, with a lot of mental activity and energy	Focused and sharp, but can be easily agitated	Relaxed and unhurried, without feeling rushed or pressured.
20) How would you describe your emotional temperament?	Anxious, sensitive, and unpredictable	Quick to anger, easily agitated, Intense and passionate	Calm, slow to anger but find it difficult to detach emotionally from situations or people
21) Taste Preference	I enjoy a variety of tastes including sweet, sour, and salty flavors. I like foods with spices and might dislike bitter tastes. I tend to prefer hot, salty, or sour drinks.	I enjoy strong, intense flavors and often crave sour, salty, and spicy tastes. I might like bitter tastes and dislike too sweet or oily foods. I tend to prefer cool or refreshing drinks.	I enjoy sweet, salty, and calorie-rich tastes that provide long-lasting energy and satisfaction. I might dislike too spicy or bitter flavors. I tend to prefer warm and sweet drinks.

### Psychometric evaluation

The 21-item BPI was subjected to MCA, LCA, and nominal-response IRT to assess latent structure, item discrimination, and score precision. Items were evaluated for model fit, information, and discrimination parameters.

Test–retest reliability was assessed using Intraclass Correlation Coefficients [ICC(3,1)] over a two-week interval. Convergent validity was evaluated against AyuSoft, a clinician-administered software tool developed by the Centre for Development of Advanced Computing (C-DAC), Pune. AyuSoft comprises an 85-item, interview and examination-based questionnaire grounded in classical Ayurvedic texts (Charaka, Sushruta, and Ashtanga Hridaya), and assigns higher weightage to physical traits than to physiological or psychological traits. Dosha proportions are derived within each domain and reported as cumulative percentages. In validation studies, AyuSoft showed strong agreement with expert clinical assessments, though its use requires trained Ayurvedic practitioners and takes approximately 45 minutes to complete ( Rotti *et al.*, 2014), and divergent validity was assessed using the Mini-IPIP, a 20-item measure of the Big Five personality traits (neuroticism, extraversion, imagination, agreeableness, conscientiousness) ( Donnellan *et al.*, 2006), with the hypothesis that BPI scores would show limited overlap with general personality constructs.divergent validity against the Mini-IPIP

### Data collection and management

Data were collected via REDCap, ensuring secure, de-identified storage, automated validation checks. To minimize response biases resulting from fixed-choice order effects, the sequence of response options was independently randomized for each participant and item using REDCap's @RANDOMORDER action tag.

### Ethics

The study was approved by the Institutional Ethics Committee, National Institute of Mental Health and Neurosciences (Ref No. NIMHANS/HECAIM/6th Meeting/2021-22) and conducted in accordance with the Declaration of Helsinki. All participants provided electronic informed consent before participating in the study. The informed consent process included a detailed information sheet explaining the study purpose, procedures, voluntary nature of participation, confidentiality measures, and contact information for queries. Participants were required to actively check a box indicating their understanding and consent before accessing the survey. No minors (individuals under 18 years) were included in the study, as verified by age eligibility screening at the beginning of the survey.

### Data analysis

Data analysis involved several stages, utilizing various R packages. MCA was performed using the FactoMineR package ( Lê *et al.*, 2008) to explore item relationships. LCA was conducted using the poLCA package ( Linzer & Lewis, 2011) to identify latent Prakriti classes. IRT analysis was performed using the mirt package ( Chalmers, 2012) to evaluate item characteristics and the reliability of each latent factor scale. Test-retest reliability was assessed using ICCs. Convergent and divergent validity were evaluated using Pearson correlations, with a Bonferroni correction applied to adjust for multiple comparisons.

## Results

### Psychometric evaluation

***Multiple Correspondence Analysis (MCA)***

**Figure 1.**
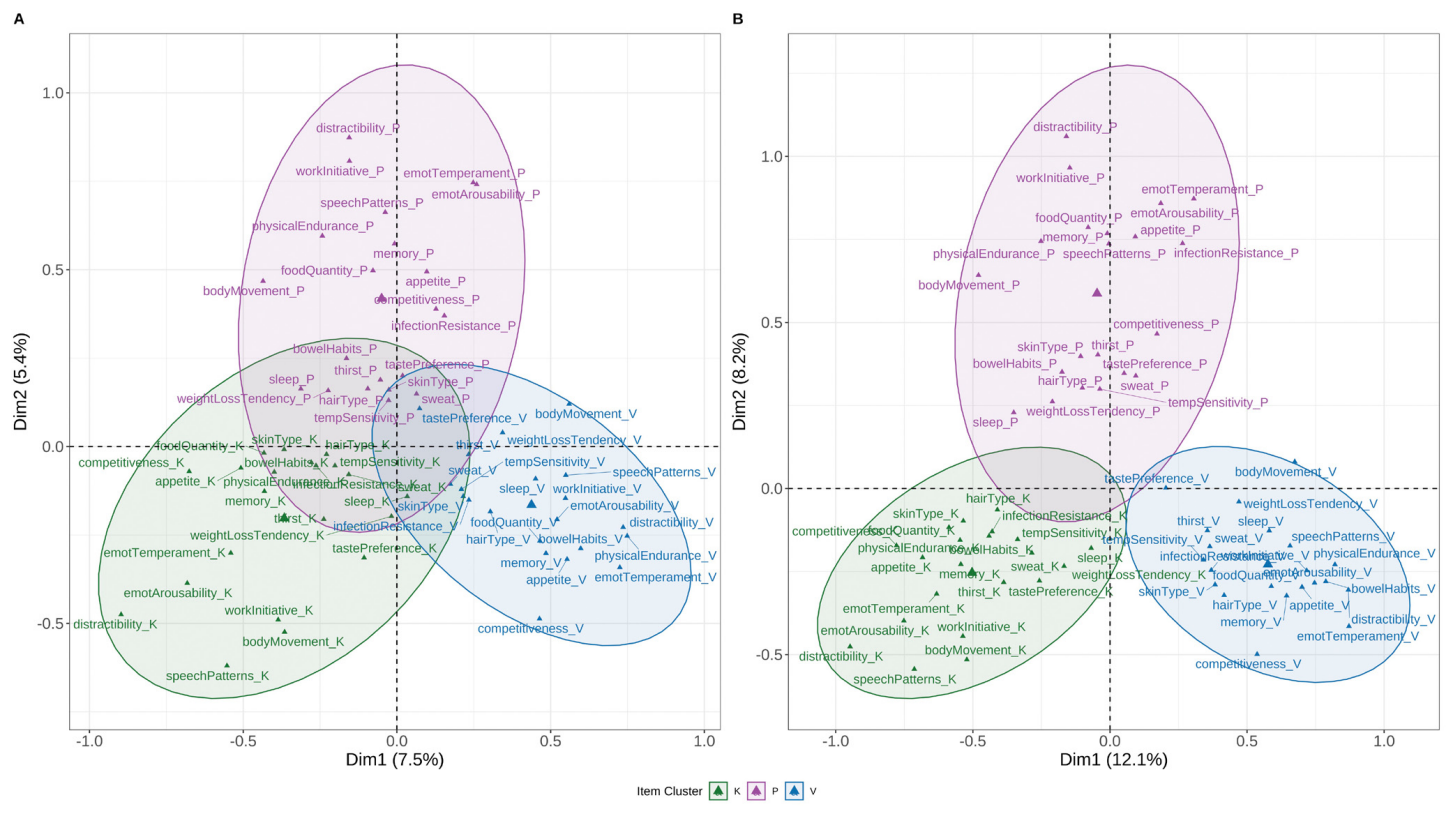
Multiple Correspondence Analysis (MCA) of BPI Items. Two-dimensional MCA plot displaying item-level clustering across the 21 items of the Brief-Prakriti Inventory. Items group distinctly along latent dosha dimensions (Vata, Pitta, Kapha), supporting the structural validity of the scale. Ellipses represent 95% confidence concentration zones for each dosha category. Axes represent the first two dimensions extracted from MCA, explaining 12.9% of total variance in the full sample and 20.3% in the extreme Prakriti subset.

MCA of the 21-item BPI in the full sample (N = 1857) revealed that the first two dimensions explained 12.9% of the variance, with three distinct clusters corresponding to dosha groupings ( Figure 1). In the extreme Prakriti subset (N = 873), variance explained increased to 20.3%, and separation between clusters was more pronounced. Items with the highest η² loadings on the first two dimensions included distractibility, emotional arousability, emotional temperament, work initiative, and speech patterns. Other high-loading items included body movement, physical endurance, appetite, food quantity, memory, and competitiveness.

***Latent Class Analysis (LCA)***

**Figure 2.**
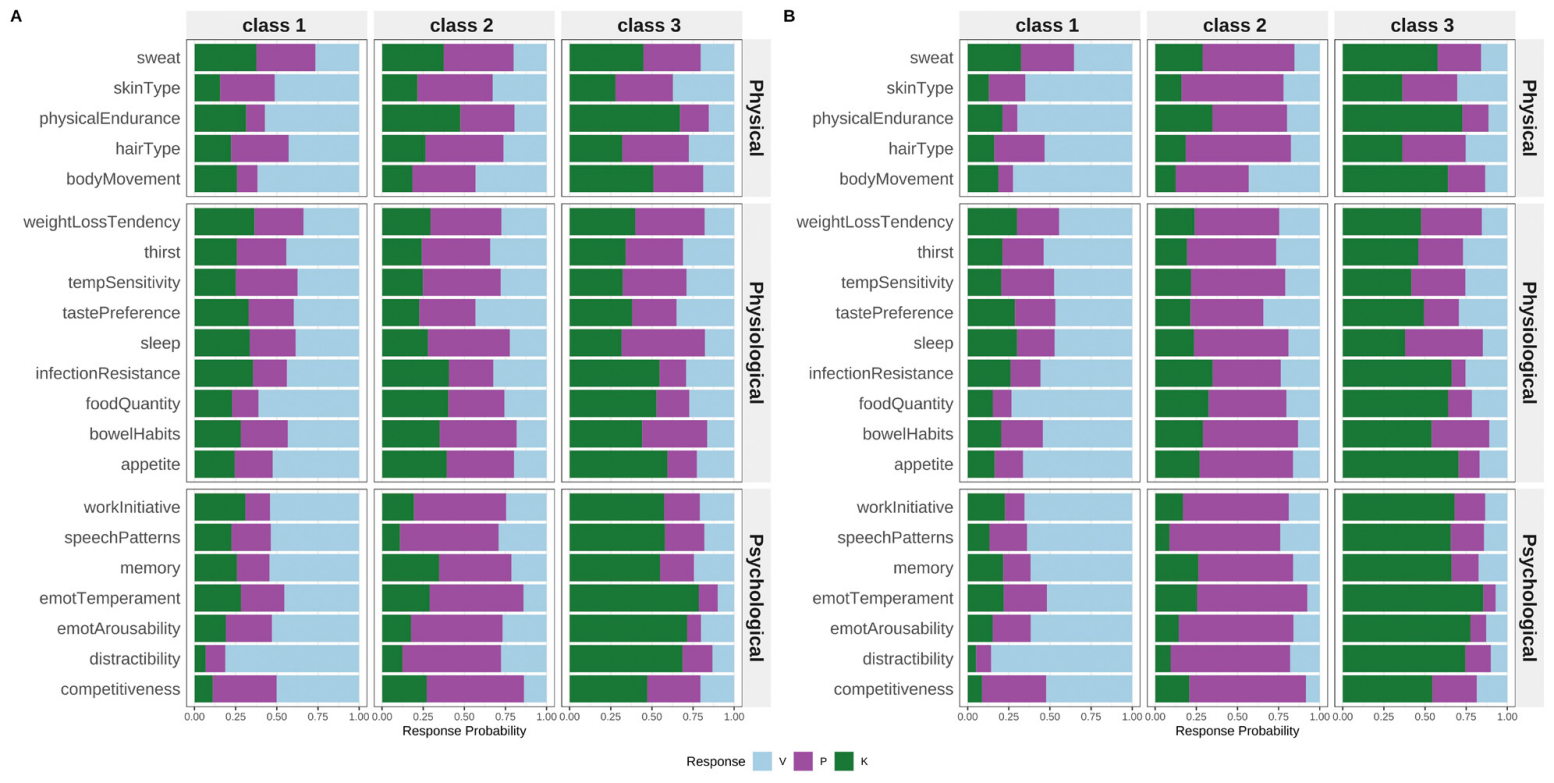
Latent Class Profiles Derived from the Brief-Prakriti Inventory. Conditional item-response probabilities across the best-fitting three-class latent class model. Each profile reflects distinct dosha dominance patterns consistent with Vata, Pitta, and Kapha typologies. Higher probabilities indicate stronger item endorsement within a latent class. The three-class solution showed optimal fit based on BIC (81,530.27 for full sample; 36,455.14 for extreme Prakriti) and entropy (R2 = 0.727 for full sample; 0.963 for extreme Prakriti).

LCA supported a three-class model in both the full sample (BIC = 81,530.27; entropy R ^2^ = 0.727) and the extreme Prakriti subset (BIC = 36,455.14; entropy R ^2^ = 0.963) ( Figure 2, Supplementary Figure 1). Class proportions were similar across samples, with item-response patterns aligning with Vata, Pitta, and Kapha characteristics. Class distinctions were sharper in the extreme Prakriti group.

***Item Response Theory (IRT)***

**Figure 3.**
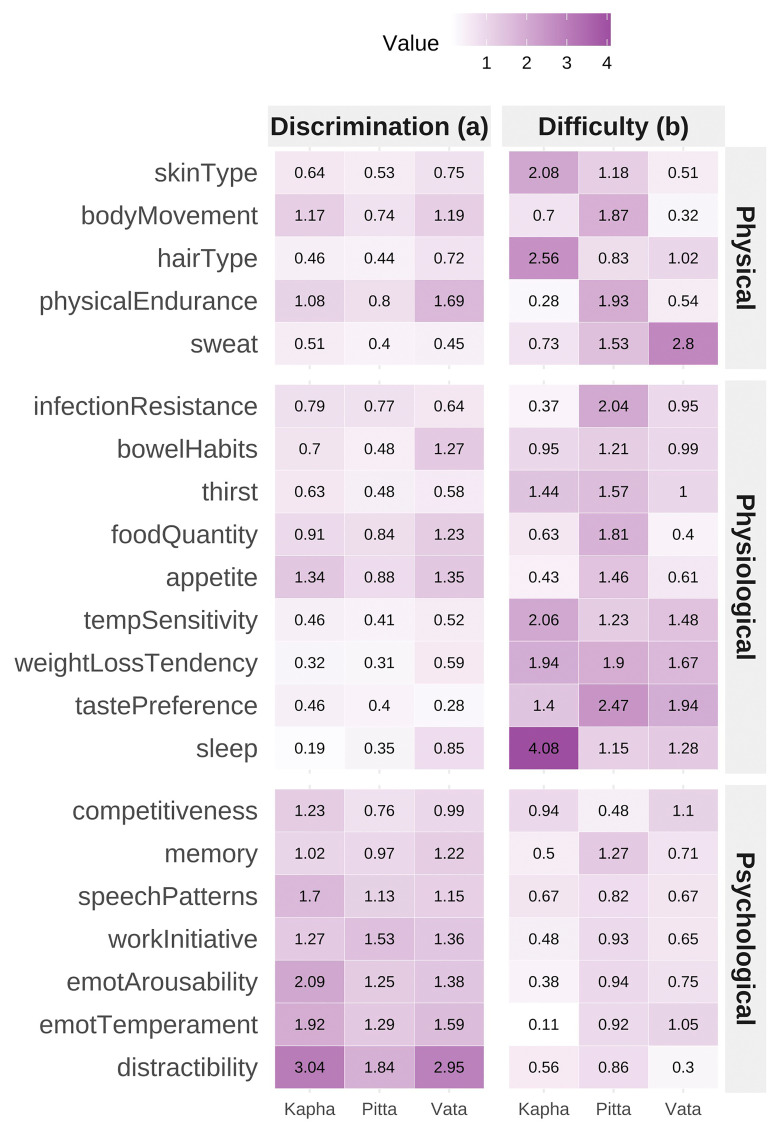
Item Response Theory (IRT) Model Parameters for BPI Items Panel displaying item characteristic curves and information functions from a graded response model fitted to the Brief-Prakriti Inventory. Curves demonstrate satisfactory item discrimination across trait levels, with psychological items showing highest discrimination parameters. Information peaks reflect item precision along the underlying dosha dimension. The three-factor IRT model showed good fit: RMSEA = 0.023, SRMSR = 0.032, TLI = 0.943, CFI = 0.967. Reliability estimates from test information functions: Vata = 0.87, Pitta = 0.75, Kapha = 0.87.

A three-factor IRT model in the full sample demonstrated good fit (RMSEA = 0.023, SRMSR = 0.032, TLI = 0.943, CFI = 0.967) ( Figure 3). Unidimensional IRT models for each dosha showed stronger fit indices in the extreme Prakriti group (CFI = 0.97–0.98) than in the full sample (CFI = 0.75–0.91). Reliability from test information functions was Vata = 0.87, Pitta = 0.75, Kapha = 0.87. Discrimination parameters were highest for psychological items, followed by physical and physiological items.

### Validity

***Test–retest reliability***

In 116 participants retested after one month, ICCs indicated good to excellent stability: Vata = 0.90 (95% CI 0.86–0.93), Pitta = 0.83 (0.76–0.88), Kapha = 0.84 (0.78–0.89).

***Convergent validity***

In 104 participants, BPI subscales correlated strongly with corresponding AYUsoft ratings: Vata r = 0.84, Pitta r = 0.78, Kapha r = 0.82 (all p < .001) (Supplementary Figure 2).

***Divergent validity***

**Figure 4.**
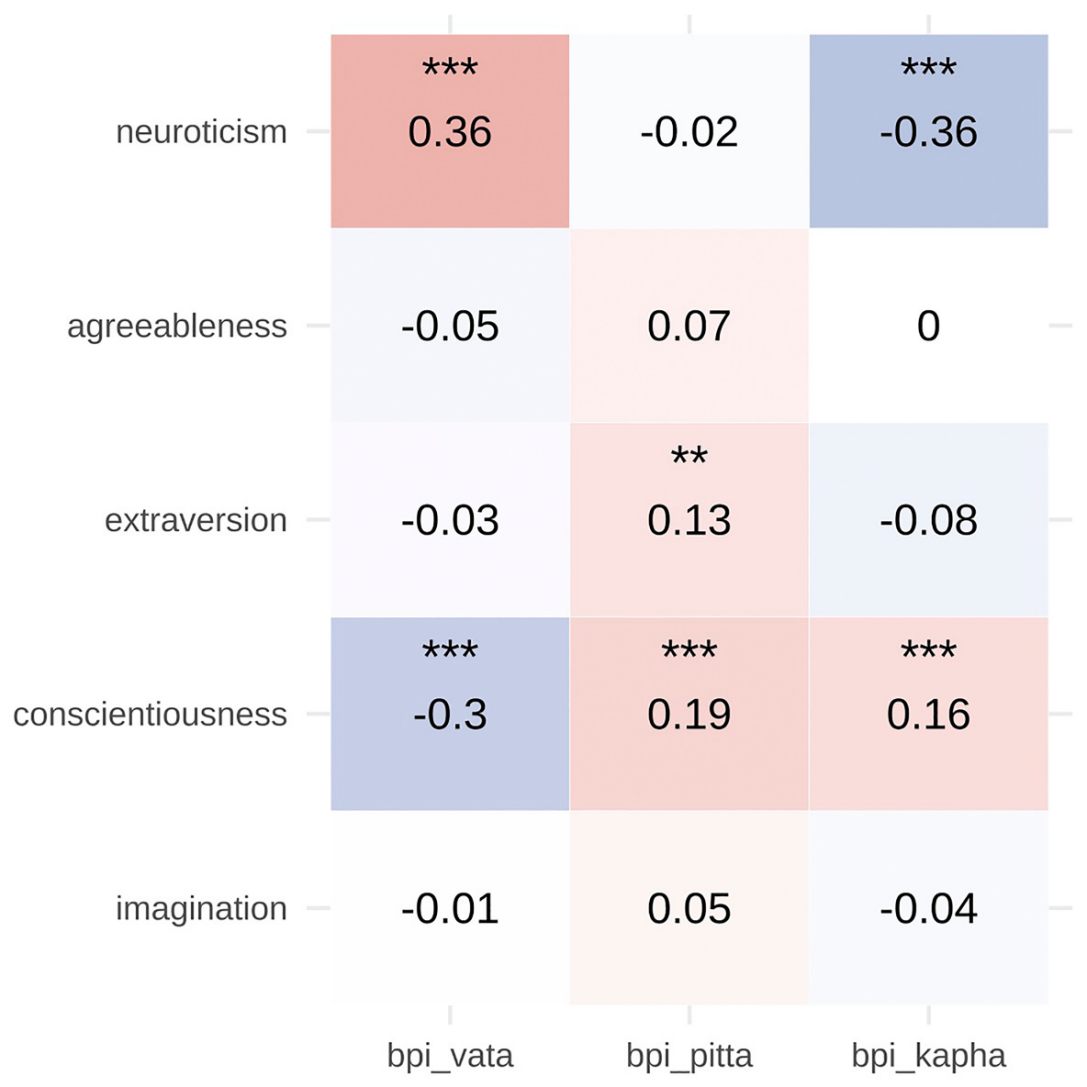
Divergent Validity of BPI with MINI-IPIP Personality Traits Scatterplots depicting the relationship between BPI dosha scores (Vata, Pitta, Kapha) and Big Five personality traits (Neuroticism, Extraversion, Imagination, Agreeableness, Conscientiousness) measured by the Mini-IPIP. The generally weak associations (most r < 0.30) support the divergent validity of the BPI, indicating that it captures constructs distinct from conventional personality dimensions. Notable associations include Vata with neuroticism (r = 0.36) and Kapha with conscientiousness (r = 0.16), suggesting some theoretical overlap while maintaining construct distinctiveness.

In 541 participants, correlations between BPI subscales and Mini-IPIP traits were generally weak ( Figure 4). Vata was positively associated with neuroticism (r = 0.36) and negatively with conscientiousness (r = –0.30). Kapha was negatively associated with neuroticism (r = –0.36) and positively with conscientiousness (r = 0.16). Pitta showed small positive associations with conscientiousness (r = 0.19) and extraversion (r = 0.13) (all p < .01).

## Discussion

The present study developed and validated the BPI, a 21-item self-report instrument designed to assess latent dosha typology through psychometrically rigorous methods. Drawing on data from a large community sample, we demonstrated that the BPI exhibits robust structural validity, satisfactory reliability, and meaningful convergent and divergent validity. Across exploratory frameworks such as multiple correspondence analysis (MCA) and latent class analysis (LCA), and confirmatory modeling using item response theory (IRT), results consistently supported a three-factor dosha model, with distinct psychological, physical, and physiological correlates.

Ayurvedic texts describe seven broad constitution types: three single-dosha extremes (Vata, Pitta, Kapha), three dual-dosha combinations (Vata–Pitta, Pitta–Kapha, Vata–Kapha), and a balanced type (Sama Prakriti). In our analyses, the extreme Prakriti subset showed the clearest separation into three latent factors. This aligns with Ayurvedic descriptions in which single-dominant constitutions manifest more prototypical features, while dual- and balanced-dosha types exhibit blended or attenuated characteristics, making empirical separation more difficult. The BPI captured these distinctions with notable precision, particularly for single-dosha types.

Recent empirical evidence provides converging support for constitution-based subgroups. In Prakriti research, genomic studies have identified distinct genetic variations associated with the traditional types. A landmark genome-wide analysis reported multiple SNP markers differing between Vata, Pitta, and Kapha dominant individuals, with signatures strong enough to cluster participants into Prakriti groups independently of ancestry ( Govindaraj *et al.*, 2015). Such findings suggest a biological basis for the Ayurvedic typology, echoing modern genomics-driven approaches to precision medicine. Beyond genetics, studies have identified inflammatory markers, metabolic pathways, and physiological traits such as cardiovascular parameters and enzyme activity across Prakriti groups ( Ghodke *et al.*, 2011; Mahalle *et al.*, 2012; Shirolkar *et al.*, 2018). Analogous evidence exists in Sasang constitutional medicine, where the four types correspond to distinct anthropometric, metabolic, and even genetic profiles ( Hur *et al.*, 2018). Psychological investigations of Sasang also reveal temperament patterns paralleling Western personality constructs, such as extraversion and neuroticism ( Chae *et al.*, 2009). Taken together, these findings imply that traditional typologies reflect reproducible multi-domain phenotypic signatures rather than pre-scientific abstractions, aligning with contemporary systems biology and stratified medicine.

Despite these advances, significant challenges remain in integrating Prakriti into evidence-based practice. Chief among these is the lack of standardization. Classical descriptions are qualitative, making operationalization into universally accepted definitions difficult ( Gupta *et al.*, 2025). Consequently, most current assessments rely on subjective questionnaires and expert judgment, which leads to variability. Inter-rater reliability among Ayurvedic physicians is modest (κ ≈ 0.2–0.4) ( Kurande *et al.*, 2013), underscoring inconsistency in clinical application. Moreover, in the absence of a gold-standard definition or objective biomarker, validating new instruments or algorithms remains difficult since no clear benchmark exists ( Edavalath & Bharathan, 2021). Addressing this gap requires development of a rigorous reference framework that combines expert consensus with measurable biological indicators, thereby enabling systematic validation of emerging tools. Parallel efforts are needed to strengthen psychometric quality, including high internal consistency, test–retest reliability, and construct validity, alongside standardized practitioner training ( Gupta *et al.*, 2025).

Against this backdrop, the BPI represents an important advance. Its strong test–retest reliability and convergence with clinician-rated AyuSoft scores ( Bhargav *et al.*, 2021) suggest its utility as a reliable and scalable self-assessment tool. At the same time, its divergence from mainstream personality traits demonstrates construct specificity. Although some overlap was observed (e.g., Pitta with extraversion, Vata with neuroticism, Kapha with conscientiousness), Prakriti represents a broader multidomain typological system distinct from Western personality models. This distinction reinforces its relevance as a complementary framework for understanding health variation.

Psychometric performance was strongest in single-dosha dominant types, with steep IRT slopes and clear class separation. This aligns with prior observations that extreme Prakriti types are the most tractable empirically and biologically, making them valuable for genotype–phenotype research ( Govindaraj *et al.*, 2015; Prasher *et al.*, 2008). Although classification of dual- and tri-dosha types remains more complex, the BPI advances measurement accuracy where construct clarity is greatest. Item-level analysis revealed that psychological dimensions contributed most strongly to dosha differentiation, while physical and physiological items showed variable discrimination, consistent with the idea that constitutional physiological traits are most evident in extreme profiles.

Several limitations warrant acknowledgment. Despite AyuSoft serving as a comparator, the absence of an objective gold standard constrains criterion validation. Validation was also restricted to an Indian community sample, raising questions about generalizability across cultures. Precision was lower for mixed- and balanced-dosha classifications, reflecting the inherent complexity of these types. Finally, predictive validity for health and clinical outcomes remains untested, necessitating longitudinal and cross-cultural research.

In conclusion, the BPI provides a reliable and valid framework for Prakriti assessment, with advantages of brevity, self-report, and scalability. Its suitability for large-scale epidemiological research and integration into clinical workflows highlights its potential translational impact. Future work should extend validation across diverse populations, establish predictive links with health outcomes, and refine the classification of mixed constitutions. Embedding the BPI into digital platforms could further advance data-driven, personalized, and preventive healthcare.

## Data Availability

Zenodo. The Brief Prakriti Inventory: Latent structure, reliability, and validity. https://doi.org/10.5281/zenodo.17453869. ( Holla & Bhargav, 2025). This project contains the following underlying data: Complete de-identified dataset containing all raw participant responses for the 21-item Brief Prakriti Inventory (BPI-21) Demographic information (age, gender, education level) Test-retest reliability subsample data Convergent validity data (BPI and AyuSoft scores) Divergent validity data (BPI and Mini-IPIP scores) R analysis scripts used for all statistical analyses including Multiple Correspondence Analysis (MCA), Latent Class Analysis (LCA), and Item Response Theory (IRT) models Extended Data: All supplementary figures and tables (Supplementary Table 1: BPI Item and Traditional Ayurvedic Reference Mapping; Supplementary Table 2: Core Guna (Qualities) Associated with Each Dosha; Supplementary Table 3: Validated Final Version of Brief-Prakriti Inventory (BPI-21) with Item-wise Response Options; Supplementary Figure 1: Model Fit Indices Across Competing Latent Class Solutions; Supplementary Figure 2: Convergent Validity of BPI with AyuSoft) Data dictionary with variable definitions and coding schemes Data is available under the terms of the Creative Commons Attribution 4.0 International (CC-BY 4.0) license. The Brief Prakriti Inventory tool is also freely accessible online at: https://redcap.link/prakriti This REDCap-based tool includes automated scoring algorithms that generate individual Prakriti profiles with percentage scores for Vata, Pitta, and Kapha doshas.
