# Supplementary material for: The Brief Prakriti Inventory: Latent structure, reliability, and validity
Source: Wellcome Open Res. 2026 Jan 16;10:669. Originally published 2025 Dec 10. [Version 2] doi: 10.12688/wellcomeopenres.25166.2 (PMC12780516; doi:10.12688/wellcomeopenres.25166.2)
Supplement: Peer review history

# Reviewer report: Reviewer response for version 2

**DOI:** 10.21956/wellcomeopenres.28450.r144901

**Published:** 2026-01-24

**Authors:** Anubha Chandla

**Affiliations:** 1 Ayurveda, CCRAS-Regional Ayurveda Research Institute, Mandi, Himachal Pradesh, India

Authors have addressed the comments adequately with required revisions and I don't have any further comments.

Is the work clearly and accurately presented and does it cite the current literature?

Yes

If applicable, is the statistical analysis and its interpretation appropriate?

I cannot comment. A qualified statistician is required.

Are all the source data underlying the results available to ensure full reproducibility?

Yes

Is the study design appropriate and is the work technically sound?

Yes

Are the conclusions drawn adequately supported by the results?

Yes

Are sufficient details of methods and analysis provided to allow replication by others?

Yes

Reviewer Expertise:

Ayurveda Researcher

I confirm that I have read this submission and believe that I have an appropriate level of expertise to confirm that it is of an acceptable scientific standard.

# Reviewer report: Reviewer response for version 1

**DOI:** 10.21956/wellcomeopenres.27740.r143882

**Published:** 2026-01-09

**Authors:** Azeem Dana

**Affiliations:** 1 School of Liberal Arts and Science, Presidency University (Ringgold ID: 484700), Bengaluru, Karnataka, India

The article describes the development and validation of the Brief Prakriti Inventory (BPI), a 21-item self-report tool designed to assess Ayurvedic constitutional types (Vata, Pitta, and Kapha). Drawing from a large community sample of 1,857 participants, the authors employed sophisticated statistical techniques, including Multiple Correspondence Analysis (MCA), Latent Class Analysis (LCA), and Item Response Theory (IRT). The study established that the BPI has strong internal and test-retest reliability, convergent validity with clinician-rated tools (AyuSoft), and divergent validity from Western "Big Five" personality traits. The authors conclude that the BPI is a reliable, open-access instrument that can bridge traditional Indian medicine with modern phenotypic research.

 The article provides a clear theoretical background, linking Prakriti to modern concepts of "cross-domain phenotypes". It cites relevant current literature on Ayurvedic genomics, machine learning applications in Prakriti, and comparative studies with Western psychology. The use of a large-scale online survey (N=1,857) provides high statistical power. The multi-stage validation strategy - moving from exploratory (MCA/LCA) to confirmatory (IRT) modelling - is technically rigorous and appropriate for categorical self-report data. The authors have followed excellent open science practices by providing the full 21-item tool, the scoring algorithm, and the R analysis scripts used for the statistical models.

 The application of nominal-response IRT is highly suitable for the BPI's response format. The interpretation of fit indices (CFI=0.967, RMSEA=0.023) is accurate and indicates a strong model fit. The underlying de-identified datasets, including demographic info and validity subsamples, are available via Zenodo. While the results strongly support the BPI’s utility for "extreme" or single-dosha types, the authors admit that the tool’s precision and the statistical separation of classes are lower for mixed- and balanced-dosha types. Since these mixed types represent a significant portion of the general population, the conclusion that the BPI is a universal framework for all Prakriti assessments is only partially supported.

 The authors should explicitly state that the current validation applies only to English-literate populations and call for future validation in regional Indian languages and non-Indian cohorts to ensure the "universal" nature of the constructs.

 Criterion Validation without a "Gold Standard": The authors correctly identify that the lack of a biological gold standard is a hurdle.

 Constructive Detail: To strengthen the conclusions, the authors could suggest specific biomarker candidates (e.g., inflammatory markers or metabolic pathways mentioned in the discussion) that should be used in follow-up studies to validate BPI scores against objective data.

 Points to Address for Scientific Soundness:

 Predictive Validity: The article currently lacks evidence that BPI scores predict clinical outcomes. To be considered "scientifically sound" for clinical journals, the authors must emphasize that the current version is a descriptive validation and that longitudinal studies are required before the tool can be used to guide treatment interventions.

 Standardized Training:As noted in the discussion, inter-rater reliability in Ayurveda is often low (κ ≈ 0.2–0.4). The authors should clarify how the self-report nature of the BPI mitigates or interacts with this known inconsistency in expert-led diagnosis.

 The sources describe the IRT model in two inconsistent ways:

 Nominal-Response IRT: In the Methods and Psychometric Evaluation sections, the authors explicitly state that they used a "nominal-response IRT" to estimate item discrimination and score precision,. This matches the description of the instrument’s design, where each item has three nominal options (Vata, Pitta, or Kapha) that participants select based on their tendencies,.

 Graded Response Model: In contrast, the caption for Figure 3 (which displays the IRT model parameters) describes the data as being from a "graded response model” fitted to the Brief-Prakriti Inventory.

 In psychometrics, these two models are mathematically distinct:

 1.  A Nominal Response Model is used for categorical data where there is no inherent order between the options (e.g., choosing between Vata, Pitta, or Kapha).

 2.  A Graded Response Model is typically used for ordered categorical data, such as Likert scales (e.g., "strongly disagree" to "strongly agree").

 Given that the BPI items are mapped to three distinct doshas rather than an ordered scale of intensity, the term "nominal-response" used in the text appears more consistent with the study's design,. However, the mention of a "graded response model" in the figure caption creates a technical inconsistency that should be clarified to ensure the manuscript is scientifically sound.

 Reproducibility of the Extreme Prakriti Subset:  The results section frequently compares the Full Sample (N=1857) with an Extreme Prakriti Subset (N=873). The results for the Extreme subset are notably better (e.g., Entropy R^2= 0.96 vs 0.72). The manuscript does not define the inclusion criteria for this Extreme subset. Was it defined by a specific score threshold (e.g., >33% difference between the dominant and second dosha)? Or was it based on the LCA posterior probabilities? Without the exact formula or cut-off score used to filter these 873 individuals, other researchers cannot replicate the study or apply this "high-precision" filter in their own work. The author may add a specific definition in the Methods section detailing how the Extreme Prakriti subset was identified (e.g., *"Participants were classified as Extreme Prakriti if their dominant Dosha score exceeded the second highest score by X points...*).

**Minor Points**

 Ipsative Data Structure: The BPI uses a "forced-choice" format (selecting Vata prevents selecting Pitta/Kapha for that item). This creates ipsative data where scores are interdependent (if Vata goes up, others must go down). While the authors used "Unidimensional IRT models for each dosha," standard IRT assumptions of local independence are often violated in forced-choice formats. A brief sentence in the Methods acknowledging this limitation or how it was handled (e.g., treating them as separate scales) would strengthen the statistical rigor.

 Divergent Validity: The finding that BPI scores have low correlation with the Big Five (Mini-IPIP) is excellent. It strongly supports the argument that Ayurveda measures a unique "psycho-somatic" construct that Western psychology misses. This should be highlighted more prominently in the Abstract.

**Conclusion**

 The study is technically impressive and represents a new standard for Ayurvedic psychometrics. The validation logic is sound, and the sample size is robust. Once the IRT model discrepancy and the Extreme Subset definition are clarified, this paper will be fully scientifically sound and a valuable addition to the literature.

Is the work clearly and accurately presented and does it cite the current literature?

Yes

If applicable, is the statistical analysis and its interpretation appropriate?

Yes

Are all the source data underlying the results available to ensure full reproducibility?

Yes

Is the study design appropriate and is the work technically sound?

Yes

Are the conclusions drawn adequately supported by the results?

Yes

Are sufficient details of methods and analysis provided to allow replication by others?

Yes

Reviewer Expertise:

Psychology, Indian Knowledge System, Health Psychology, Indian Psychology, Spiritual Psychology, Counseling, Research, Teaching and Education.

I confirm that I have read this submission and believe that I have an appropriate level of expertise to confirm that it is of an acceptable scientific standard.

# Response

**Published:** 2026-01-12

**Authors:** Hemant Bhargav

**Affiliations:** Integrative Medicine, National Institute of Mental Health and Neurosciences, Bengaluru, India, Bengaluru, Karnataka, India

We thank the reviewer for valuable comments. We have now added the following section in the manuscript: 

 Prakriti Classification Strategy and Rationale for Extreme-Group Analyses Prakriti classification was based on relative Vata, Pitta, and Kapha scores. A single extreme-dosha Prakriti was assigned when one dosha score exceeded the combined scores of the other two. Sama Prakriti was defined by all three dosha scores falling within a mid-range band (28–38), indicating constitutional balance. All remaining individuals were classified as dual-dosha Prakriti by identifying the lowest-scoring dosha and assigning the remaining two as the dominant pair (Vata–Pitta, Vata–Kapha, or Pitta–Kapha), ensuring mutually exclusive classification. Dual-dosha and Sama Prakriti profiles are expected and normative in population-based samples and reflect constitutional heterogeneity rather than limitations of the scale. Analyses were therefore conducted in both the full sample and an extreme Prakriti subset to assess robustness and discriminative validity across the full spectrum of dosha expression. The extreme Prakriti group, characterized by reduced dosha overlap, was expected to show clearer structural separation and stronger measurement properties. Comparisons across the full and extreme samples allowed evaluation of scale performance under typical and maximal trait expression, a standard psychometric approach to examine sensitivity, boundary validity, and theoretical alignment. We have also corrected the typographical error in the Figure 3 legend.

# Reviewer report: Reviewer response for version 1

**DOI:** 10.21956/wellcomeopenres.27740.r142080

**Published:** 2026-01-08

**Authors:** Anubha Chandla

**Affiliations:** 1 Ayurveda, CCRAS-Regional Ayurveda Research Institute, Mandi, Himachal Pradesh, India

The manuscript presents an important work on the development of a validated and self-reported Ayurvedic Prakriti assessment tool (BPI).

 After reviewing the manuscript and supplementary files, I would say that the work is novel, and each section of the article is well-written in all aspects, from the relevance of the work to the methodology, results, and interpretation. 

 The study clearly states its limitations and also outlines its future scope.

 My comments are:

 1.  What was the criteria (percentage range) to determine the prakriti type and how the cut off was decided. After going through the supplementary data files of study participants, it looks >50% for a dosha was considered dominant prakriti. But in a case where v=51%, P=44%, K=5% interpreting prakriti as vataj doesn't seem appropriate!

 2.  The study says that psychometric performance was strongest in single dosha dominant types. However, based on practical experience and according to Ayurveda dwandaja (dual) doshaj prakriti individuals are most commonly seen. Which may restrict the  practical use of the tool.

 3. To analyze the statistical content, I recommend consulting a biostatistician.

Is the work clearly and accurately presented and does it cite the current literature?

Yes

If applicable, is the statistical analysis and its interpretation appropriate?

I cannot comment. A qualified statistician is required.

Are all the source data underlying the results available to ensure full reproducibility?

Yes

Is the study design appropriate and is the work technically sound?

Yes

Are the conclusions drawn adequately supported by the results?

Yes

Are sufficient details of methods and analysis provided to allow replication by others?

Yes

Reviewer Expertise:

Ayurveda Researcher

I confirm that I have read this submission and believe that I have an appropriate level of expertise to confirm that it is of an acceptable scientific standard, however I have significant reservations, as outlined above.

# Response

**Published:** 2026-01-10

**Authors:** Hemant Bhargav

**Affiliations:** Integrative Medicine, National Institute of Mental Health and Neurosciences, Bengaluru, India, Bengaluru, Karnataka, India

We thank the reviewer for the positive assessment. As noted in the response to Reviewer 2 (Comment 2), an explicit operational definition was used in this study to identify extreme Prakriti profiles for psychometric evaluation. This definition represents one principled approach to operationalizing dosha dominance. Future studies may adopt alternative or more stringent definitions of extreme Prakriti tailored to specific clinical, biological, or mechanistic research questions.

# Reviewer report: Reviewer response for version 1

**DOI:** 10.21956/wellcomeopenres.27740.r143877

**Published:** 2026-01-07

**Authors:** Sumeet Goel

**Affiliations:** 1 All India Institute of Ayurveda, Goa, Goa, India

**1) Is the work clearly and accurately presented and does it cite the current literature?**

**Partly.**

 The manuscript is generally clear and well-structured, with up-to-date contextualization that includes recent work on standardisation and psychometrics in Prakriti assessment. However, a few internal inconsistencies (e.g., test–retest interval described differently in Methods vs Results) and some terminology/model-description issues (IRT “graded response” wording for nominal categories) require correction/clarification to maintain technical accuracy.

**2) Is the study design appropriate and does the work have academic merit?**

**Yes.**

 A large sample size and multi-method latent structure approach (MCA + LCA + IRT) is appropriate for scale development, with the motivation being an openly described, brief, psychometrically evaluated Prakriti instrument of clear academic merit for integrative medicine and cross-cultural personality/phenotyping research.

**3) Are sufficient details of methods and analysis provided to allow replication by others?**

**Partly.**

 Replication is strongly supported by sharing data and scripts, but key operational details should be explicitly stated in the manuscript (not only in code/supplements), including: how “extreme Prakriti” was defined; how dominant vs mixed types are classified from raw scores; which exact IRT nominal model specification was used; handling of missingness beyond exclusion thresholds; and whether any sample-splitting or cross-validation was used.

**4) If applicable, is the statistical analysis and its interpretation appropriate?**

**Partly.**

 The overall analytic strategy is appropriate for categorical item responses and typological structure; fit indices and reliability reporting are helpful. That said, the manuscript's description of the IRT model appears internally inconsistent (nominal options keyed to doshas vs “graded response model" language), and interpretation should more explicitly acknowledge the psychometric implications of forced-choice, dosha-keyed response formats.

**5) Are all the source data underlying the results available to ensure full reproducibility?**

**Yes.**

**6) Are the conclusions drawn adequately supported by the results?**

**Yes**

**also**

**1. Clarify the IRT model specification (nominal vs graded/ordinal) and align text/figure captions accordingly.**

 Each item forces selection of one of three **nominal** options mapped to Vata/Pitta/Kapha. The manuscript/figure captions use phrasing suggestive of a **graded response model**, which is typically for ordinal Likert-type categories. Please state explicitly:

- which IRT model was fitted (e.g., **nominal response model** / multinomial IRT),
- whether the three-factor model was a multidimensional nominal model,
- and how item parameters were interpreted under that model.
  This is essential for readers to evaluate discrimination/difficulty interpretations appropriately.

**2. Define “extreme Prakriti subset” and classification thresholds in the manuscript.**

 Several key findings rely on the “extreme” subset (N = 873), which provides improved separation/fit. Please specify:

- the exact algorithm/threshold used to label a participant “extreme” (e.g., ≥X% in one dosha; or a minimum margin over the second-highest dosha),
- how many participants were classified into each extreme dosha group,
- and whether thresholds were pre-specified or data-driven.
  Similarly, please describe how mixed (dual/tri-dosha) and “Sama” classifications are handled, if at all, since the Discussion references these categories.

Is the work clearly and accurately presented and does it cite the current literature?

Partly

If applicable, is the statistical analysis and its interpretation appropriate?

Yes

Are all the source data underlying the results available to ensure full reproducibility?

Yes

Is the study design appropriate and is the work technically sound?

Yes

Are the conclusions drawn adequately supported by the results?

Yes

Are sufficient details of methods and analysis provided to allow replication by others?

Partly

Reviewer Expertise:

Ayurveda (Paediatrics in Ayurveda, Observational studies, RCTs), Systematic Reviews, Scoping reviews, Policy studies on IPR in TK, Documentation, AI based biopiracy, Benefit Sharing.

I confirm that I have read this submission and believe that I have an appropriate level of expertise to confirm that it is of an acceptable scientific standard.

# Response

**Published:** 2026-01-10

**Authors:** Hemant Bhargav

**Affiliations:** Integrative Medicine, National Institute of Mental Health and Neurosciences, Bengaluru, India, Bengaluru, Karnataka, India

We thank the reviewer for the positive assessment.

**C1.** IRT model specification

**Response:** Each BPI-21 item uses three nominal response options (Vata, Pitta, Kapha); accordingly, nominal response IRT models were fitted using the mirt package, and figure captions have been revised to reflect nominal IRT terminology rather than graded or ordinal models. A multidimensional nominal IRT model with three latent factors (Vata, Pitta, Kapha) was first fitted to the full sample to evaluate overall model fit and latent structure. Subsequently, separate unidimensional nominal IRT models were fitted for each dosha, with responses coded as 1 for the target dosha and 0 for the other two, to estimate item discrimination and measurement precision; full analysis scripts are publicly available on Zenodo, and improved fit was observed in the extreme Prakriti subset.

**C2.** Extreme Prakriti subset definition

**Response: **The extreme Prakriti subset was defined a priori using an explicit rule-based criterion of relative dosha dominance to minimize overlap, and group sizes are reported in the manuscript. Dual-dosha and Sama profiles were retained in full-sample analyses as normative categories, while the extreme subset was used to evaluate discriminative validity under maximal trait expression.

# Reviewer report: Reviewer response for version 1

**DOI:** 10.21956/wellcomeopenres.27740.r142089

**Published:** 2026-01-07

**Authors:** Remya E

**Affiliations:** 1 Central Ayurveda Research Institute Bhubaneswar, Bhubaneswar, India

Thank you for the opportunity to review this article on Brief Prakriti Inventory.

 Revising the area of item development and content validity will increase the scientific soundness of the study. *The manuscript states that items were derived from classical texts and expert consensus, but **specific procedures are not described*** ***.*** *Specify *which texts*, *how constructs were operationalized*, *number and expertise of reviewers*, and whether any formal content-validity indexing (e.g., CVI) was performed.*

 The “extreme Prakriti subset” plays a central role in the psychometric results, yet selection criteria are insufficiently detailed.

Is the work clearly and accurately presented and does it cite the current literature?

Yes

If applicable, is the statistical analysis and its interpretation appropriate?

I cannot comment. A qualified statistician is required.

Are all the source data underlying the results available to ensure full reproducibility?

No

Is the study design appropriate and is the work technically sound?

Yes

Are the conclusions drawn adequately supported by the results?

Yes

Are sufficient details of methods and analysis provided to allow replication by others?

Yes

Reviewer Expertise:

NA

I confirm that I have read this submission and believe that I have an appropriate level of expertise to confirm that it is of an acceptable scientific standard, however I have significant reservations, as outlined above.

# Response

**Published:** 2026-01-10

**Authors:** Hemant Bhargav

**Affiliations:** Integrative Medicine, National Institute of Mental Health and Neurosciences, Bengaluru, India, Bengaluru, Karnataka, India

We thank the reviewer for the constructive comments and address the key concerns below.

**C1: Item development and content validity**

 Response: Details of item development and content validity are provided in the Supplementary Information. The initial 30-item pool was derived from classical Ayurvedic texts, with explicit item-to-text mapping and dosha attribution (Supplementary Table 1) and core guna associations (Supplementary Table 2). Item retention and reduction to the final 21-item BPI were guided by psychometric validation using MCA, LCA, and IRT, rather than formal content validity indexing. This approach has been clarified in the manuscript.

**C2: Extreme Prakriti subset**

 Response: Dual-dosha and Sama Prakriti profiles are expected and normative in population-based samples and do not reflect scale limitation. The extreme Prakriti subset was defined using explicit operational criteria to minimize dosha overlap and to evaluate discriminative validity under conditions of maximal trait expression, and the manuscript has been updated to clarify this rationale. The scale can also be used to identify dominant Prakriti profiles, which is particularly relevant for hypothesis-driven studies examining associations between dosha dominance and biological, physiological, or clinical measures. The operational definition of extreme Prakriti used here represents one principled approach; future studies may adopt alternative or more stringent definitions tailored to specific research questions. Changes: 

 "Prakriti Classification Strategy and Rationale for Extreme-Group Analyses

 Prakriti classification was based on relative Vata, Pitta, and Kapha scores. A single extreme-dosha Prakriti was assigned when one dosha score exceeded the combined scores of the other two. Sama Prakriti was defined by all three dosha scores falling within a mid-range band (28–38), indicating constitutional balance. All remaining individuals were classified as dual-dosha Prakriti by identifying the lowest-scoring dosha and assigning the remaining two as the dominant pair (Vata–Pitta, Vata–Kapha, or Pitta–Kapha), ensuring mutually exclusive classification. 

 Dual-dosha and Sama Prakriti profiles are expected and normative in population-based samples and reflect constitutional heterogeneity rather than limitations of the scale. Analyses were therefore conducted in both the full sample and an extreme Prakriti subset to assess robustness and discriminative validity across the full spectrum of dosha expression. The extreme Prakriti group, characterized by reduced dosha overlap, was expected to show clearer structural separation and stronger measurement properties. Comparisons across the full and extreme samples allowed evaluation of scale performance under typical and maximal trait expression, a standard psychometric approach to examine sensitivity, boundary validity, and theoretical alignment."

**C3: Data availability**

 Response: All underlying source data are publicly available via Zenodo (DOI: 10.5281/zenodo.17453869), including the complete de-identified dataset, analysis scripts and the supplementary information. The BPI-21 is also freely accessible as a REDCap-based tool with automated scoring. This is documented in the Data Availability Statement.

# Reviewer report: Reviewer response for version 1

**DOI:** 10.21956/wellcomeopenres.27740.r142083

**Published:** 2025-12-31

**Authors:** Praveen K

**Affiliations:** 1 Kamineni Institute of Medical Sciences, Hyderabad, India

This manuscript represents a significant and timely contribution to the field of integrative medicine and psychometric research by providing the first rigorously validated, brief self-report instrument for assessing Ayurvedic constitutional types. The authors demonstrate commendable methodological rigor through their comprehensive validation strategy, employing multiple complementary analytical approaches including Multiple Correspondence Analysis, Latent Class Analysis, and Item Response Theory—a combination that is rarely seen in traditional medicine research and sets a high standard for future instrument development in this domain. The large sample size of 1,857 participants substantially exceeds the minimum requirements for IRT analyses, lending considerable statistical power and credibility to the findings. The strong psychometric properties reported, including excellent test-retest reliability (ICCs 0.83–0.90), robust convergent validity with clinician-administered AyuSoft assessments (r = 0.78–0.84), and importantly, the demonstration of divergent validity against Western personality constructs, collectively establish the BPI as both reliable and measuring a distinct construct. The authors' transparent acknowledgment of limitations, particularly regarding the absence of a gold standard and the need for cross-cultural validation, reflects scientific integrity and appropriately contextualizes the findings. The decision to make the instrument freely accessible via REDCap and to deposit all data and analysis scripts in a public repository exemplifies open science practices that will facilitate replication and extension of this work. This paper successfully bridges traditional knowledge systems with contemporary psychometric standards and will serve as a valuable resource for researchers investigating personalized and culturally-informed approaches to health and wellbeing.

Is the work clearly and accurately presented and does it cite the current literature?

Yes

If applicable, is the statistical analysis and its interpretation appropriate?

Yes

Are all the source data underlying the results available to ensure full reproducibility?

Yes

Is the study design appropriate and is the work technically sound?

Yes

Are the conclusions drawn adequately supported by the results?

Yes

Are sufficient details of methods and analysis provided to allow replication by others?

Yes

Reviewer Expertise:

Psychiatry

I confirm that I have read this submission and believe that I have an appropriate level of expertise to confirm that it is of an acceptable scientific standard.
